# Supplementary figures and images for: Regeneration of duckweed (Lemna turonifera) involves genetic molecular regulation and cyclohexane release
Source: PLoS One. 2022 Jan 6;17(1):e0254265. doi: 10.1371/journal.pone.0254265 (PMC8735602; doi:10.1371/journal.pone.0254265)

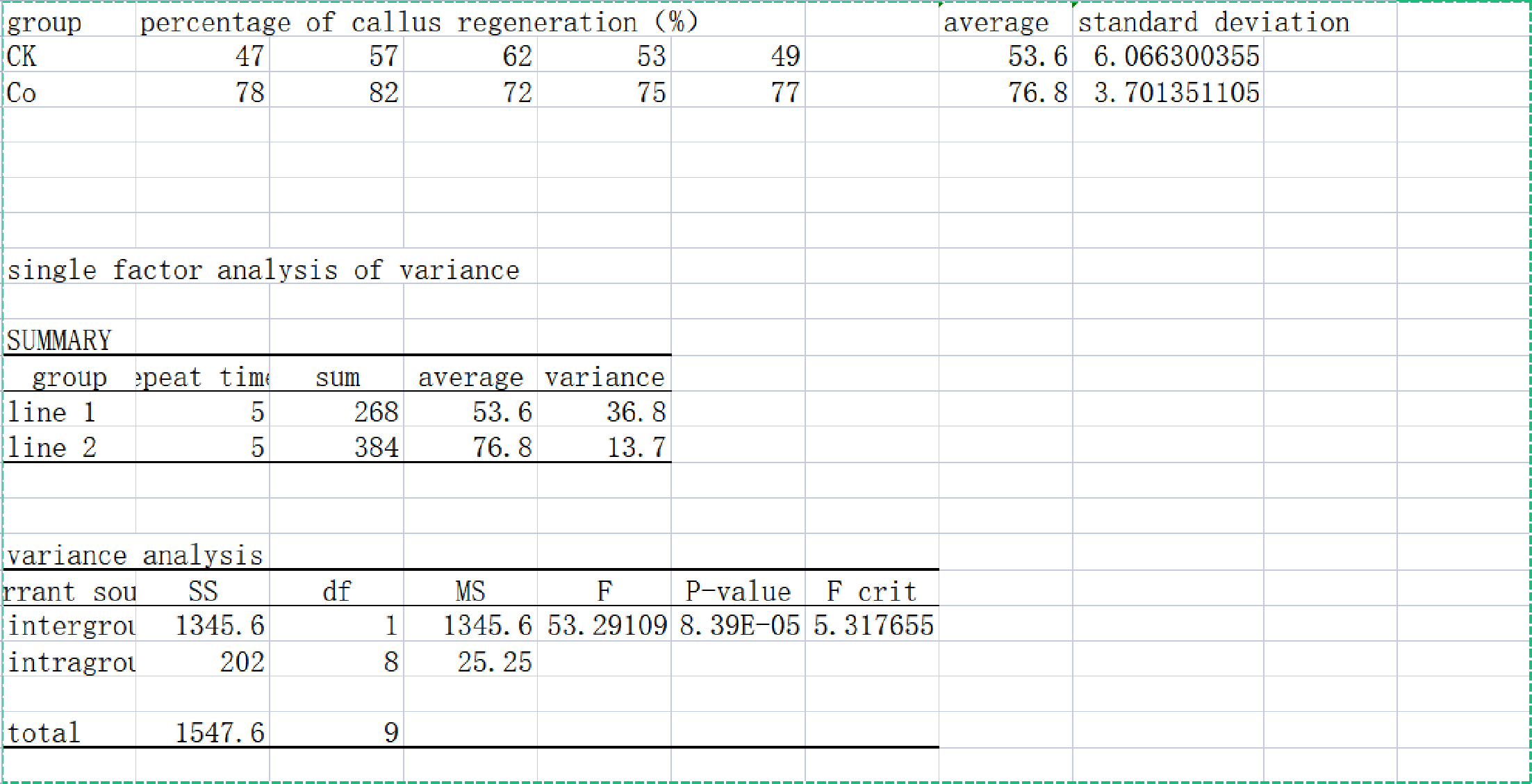

Supplement: S1 Fig — CK, control treatment; Co, the callus co-cultured with regenerating callus. (TIFF) [file pone.0254265.s001.tiff]

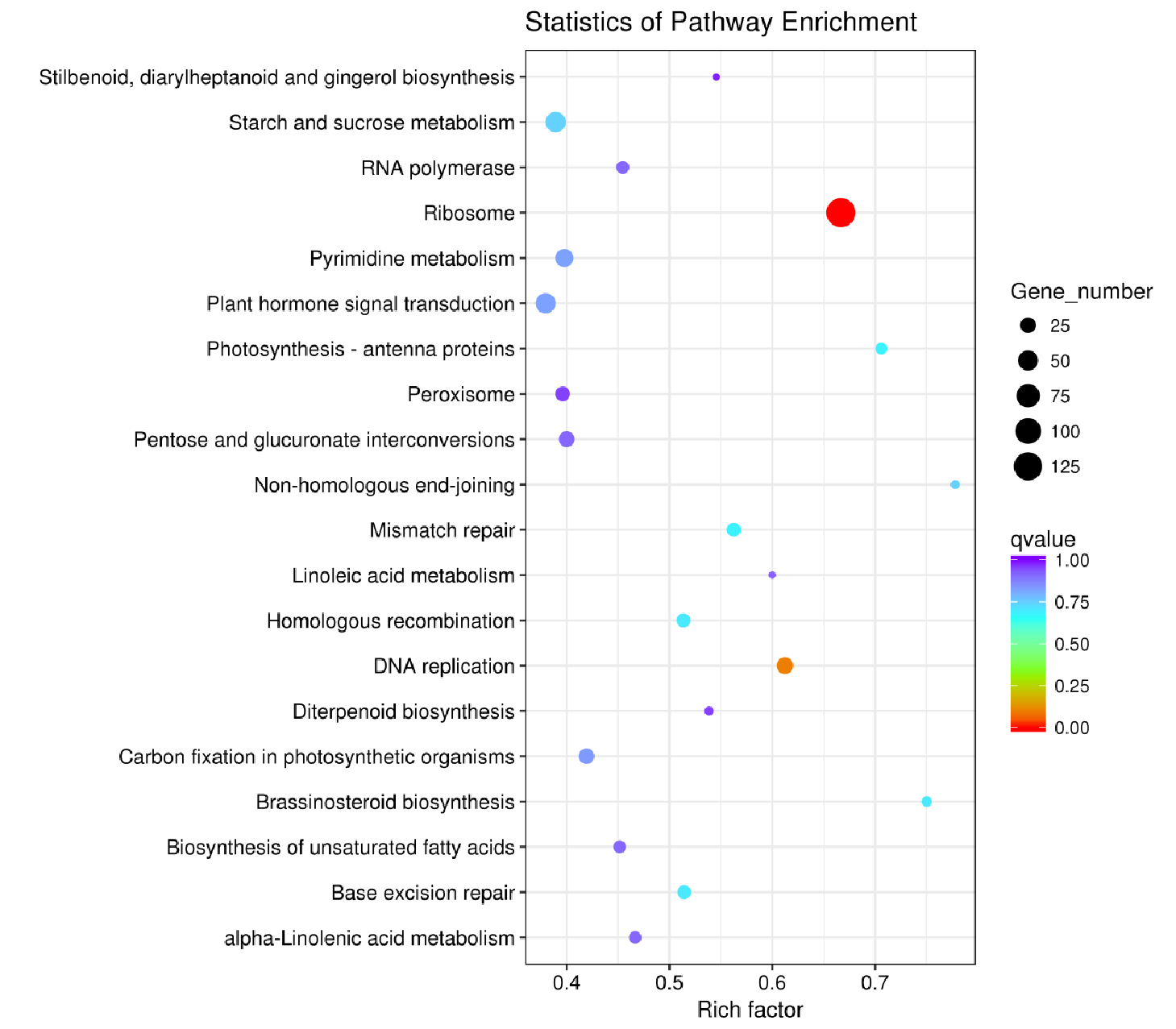

Supplement: S2 Fig — The 20 pathways with the most signifcant enrichment of ‘RG vs CL’. (TIFF) [file pone.0254265.s002.tiff]

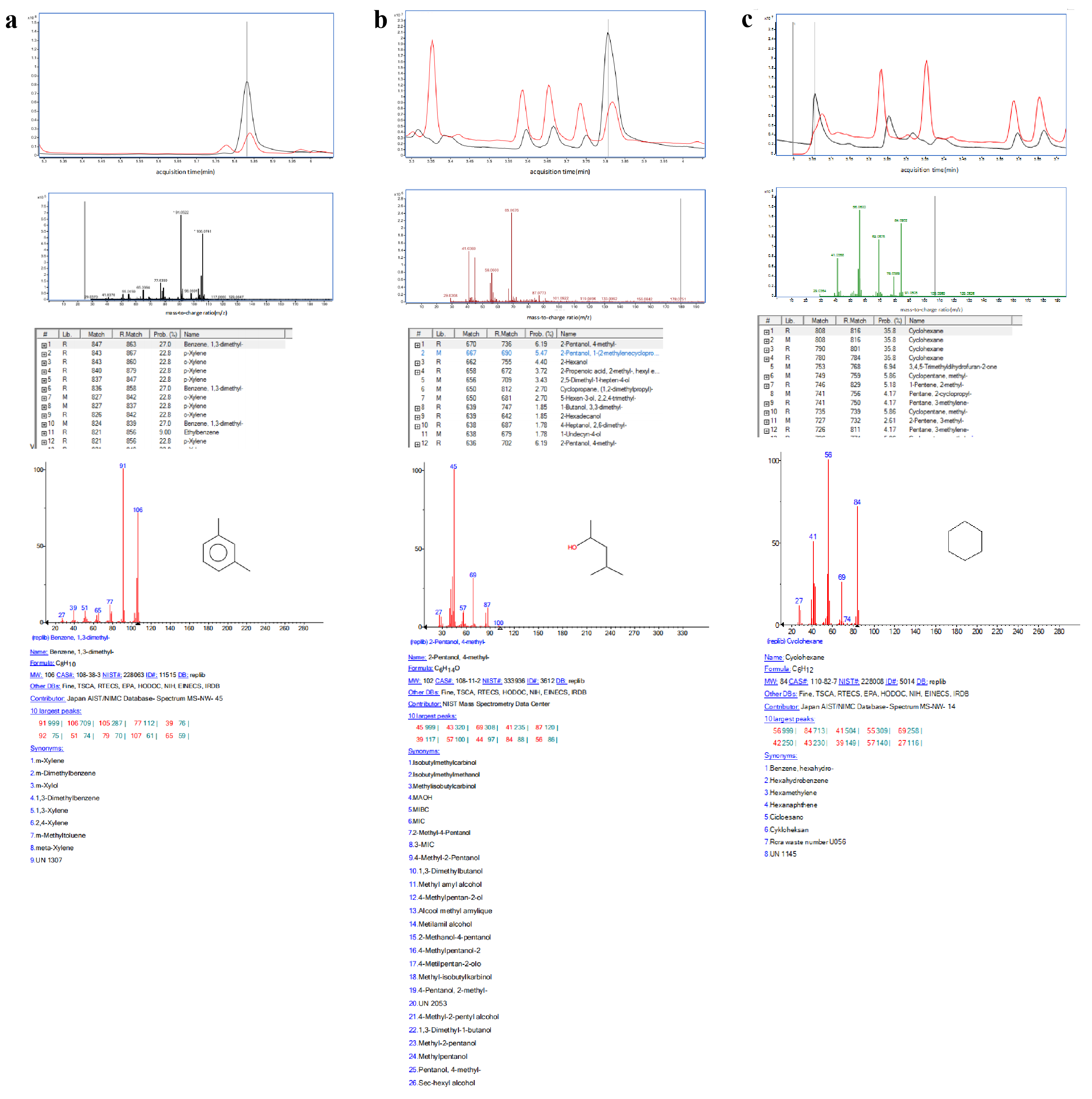

Supplement: S3 Fig — a Data analyze of 1, 3-dimethyl benzene. b Data analyze of 4-methyl-2-pentanol. c Data analyze of cyclohexane. (TIFF) [file pone.0254265.s003.tiff]

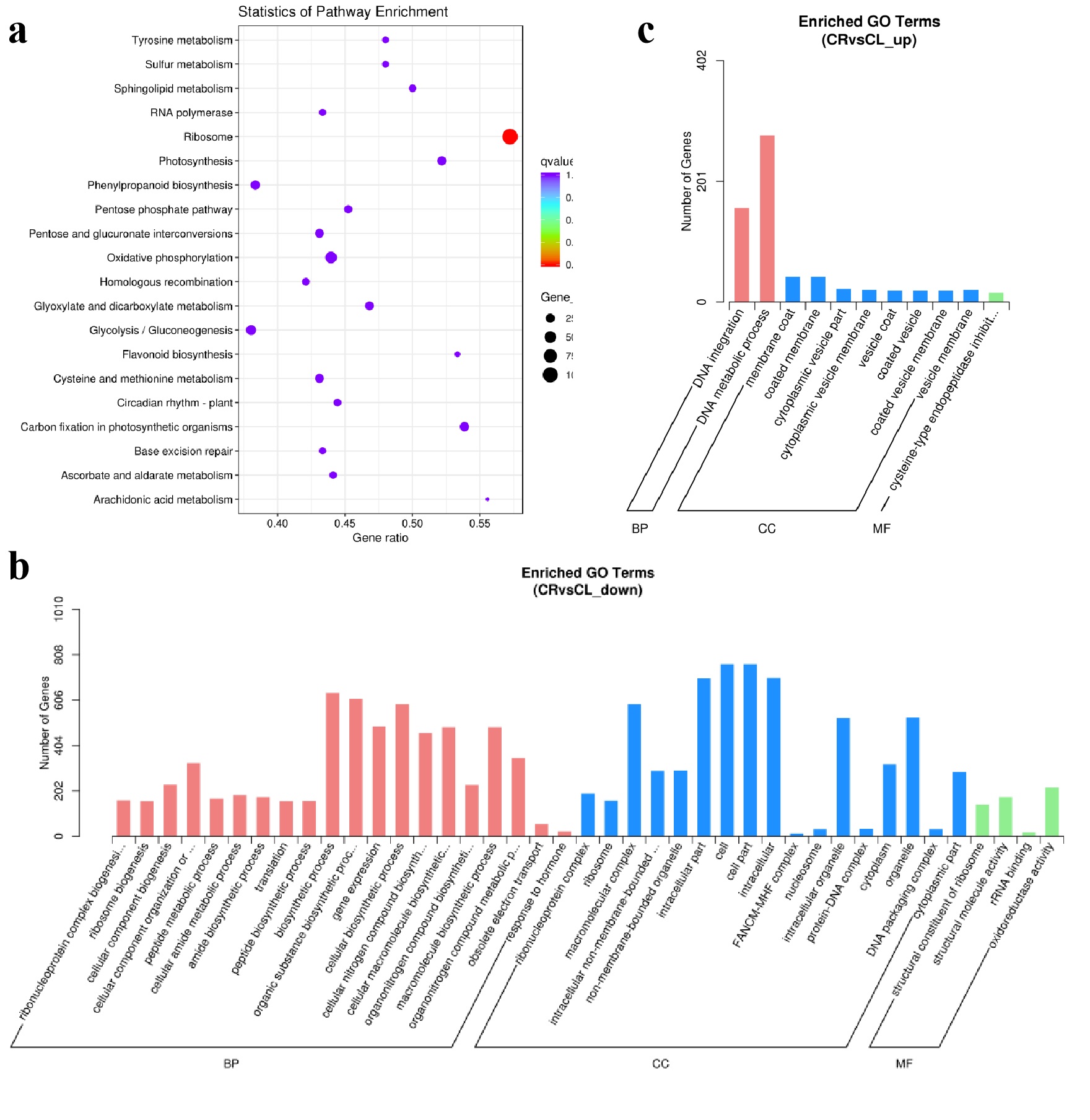

Supplement: S4 Fig — a The 20 pathways with the most signifcant enrichment of ‘CRvs CL’. b Histogram of GO enrichment of diferential genes in ‘CR vs CL’ with the most down-regulated DEGs. c Histogram of GO enrichment of diferential genes in ‘CR vs CL’ with the most up-regulated DEGs. (TIFF) [file pone.0254265.s004.tiff]

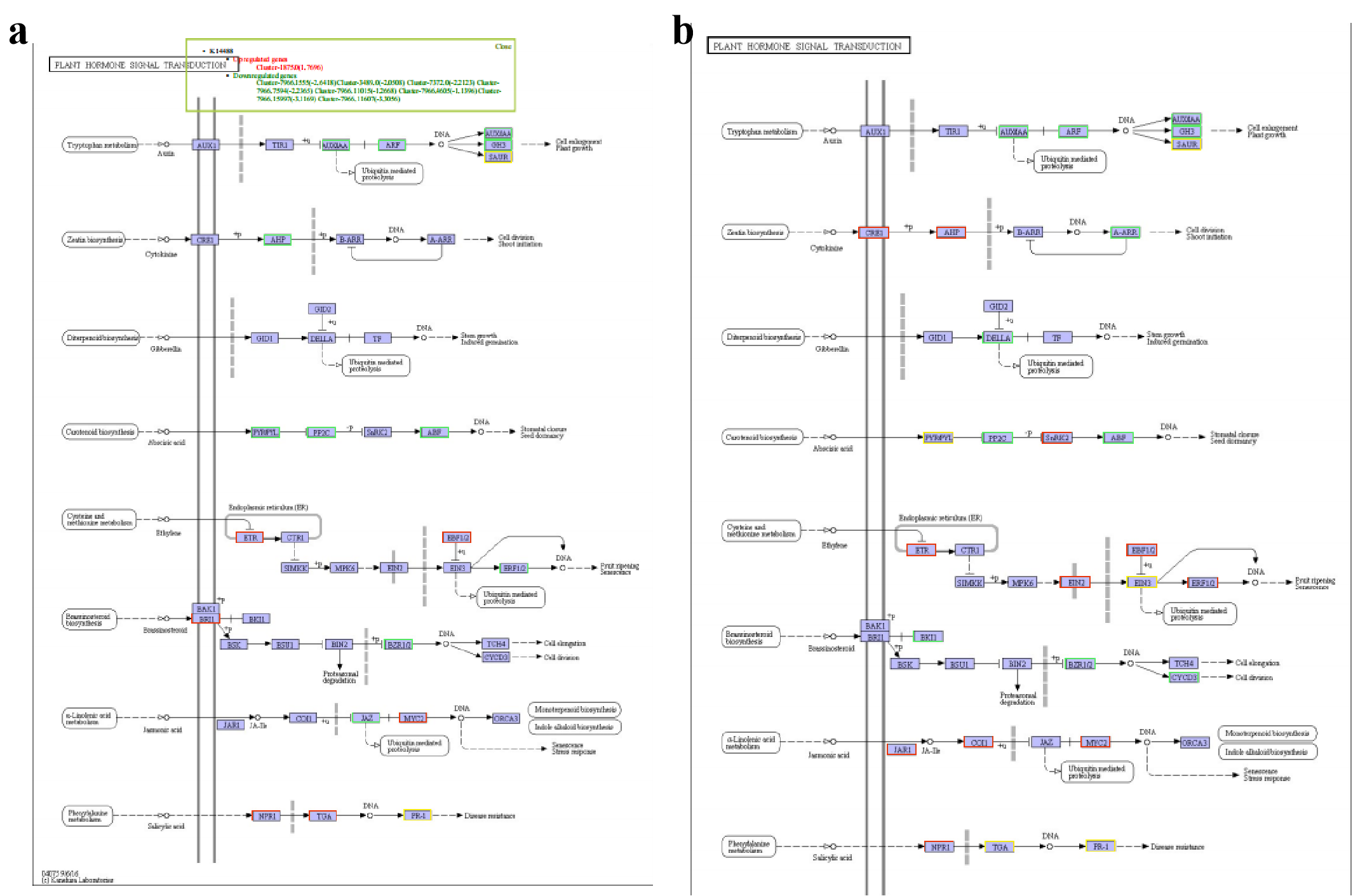

Supplement: S5 Fig — a The expression of genes related to hormones in regenerating callus vs callus. b The expression of genes related to hormones in callus treated with cyclohexane vs callus. (TIFF) [file pone.0254265.s005.tiff]
