# Supplementary material for: Regeneration of duckweed (Lemna turonifera) involves genetic molecular regulation and cyclohexane release
Source: PLoS One. 2022 Jan 6;17(1):e0254265. doi: 10.1371/journal.pone.0254265 (PMC8735602; doi:10.1371/journal.pone.0254265)
Supplement: Attachment — Submitted filename: Comments to PONE-D-21-17629.pdf [file pone.0254265.s006.pdf]

Recommendation to PONE-D-21-17629

Comments to the Authors

1. The motivation of the study is interesting, however, the hypothesis is not clearly described. In Line 263, the hypothesis is not an accurate hypothesis.
2. In the manuscript, 'genetic transcript regulation' of the title is not proper, because there is no temporal comparison of the transcript level or upstream/downstream relationship of gene expression to explain the transcriptional regulation.
3. There is no clear description in the Materials & Methods.
   - Please define the plant materials of regenerating callus and callus.
   - In "VOCs Collection and analysis", the material quantity used for VOC collection and analysis and the analysis methods for quantitative data.
   - There is no description about RNA isolation.
   - Before "Sequencing data filtering and transcript assembly", there is no description about RNA sequencing. Please describe the method clearly, including the plant materials and the gene expression comparison between RG and CL or Cyclohexane and CL.
   - There is no description about the application of VOC on callus, including cyclohexane, 4-methyl-2-pentanol and 1, 3-dimethyl benzene. The dosage? The quantity of callus in the treatment?...
4. The callus induction medium (2 mg/L BA) is different from the formula (1 mg/L BA) in the cited reference 35 and 36, please explain.
5. Title of Table 2, 3, 4 and Fig. 3, 4, 6 needs to be revised. For example, Table 2. Gene expression in plant regeneration of Auxin. What is "plant regeneration of auxin"? Please revised them.
6. Line 150-152, please check the exact fold change of cyclohexane.
7. Line 218, Genome?. There is no study of genome sequencing in this study.
8. There is no scale bar shown in Fig. 1a and Fig. 6.
9. Line 255 and Line 261, "the regeneration of callus" and "the root formation". Exact morphological change of callus treated with cyclohexane is the root differentiation or induction from callus in this study. The term "the regeneration of callus" may be ambiguous with "the plant regeneration from callus". Please define and specify the different terms in the study.
10. I sincerely suggested that the authors use a professional editing service to avoid mistakes in English scientific writing and typing error.
    - Line 27, lower case of G in Genetic
    - Line 56-58, the sentences are not complete. vitro-> in vitro; novo-> de novo;

incubate->incubated; callus-> callus induction….

- Line 95, wrong spelling of repaire
- Line 285, formated?
- Line 245, and?
