# Supplementary material for: Regeneration of duckweed (Lemna turonifera) involves genetic molecular regulation and cyclohexane release
Source: PLoS One. 2022 Jan 6;17(1):e0254265. doi: 10.1371/journal.pone.0254265 (PMC8735602; doi:10.1371/journal.pone.0254265)
Supplement: Attachment — Submitted filename: respond.doc [file pone.0254265.s007.doc]

Dear editor：

Thank you very much for your kindly help in processing the review of our manuscript. We have carefully read these thoughtful comments from you and reviews, and the suggestions are constructive.

1. PLOS requires an ORCID iD for the corresponding author in Editorial Manager on papers submitted after December 6th, 2016. Please ensure that you have an ORCID iD and that it is validated in Editorial Manager. To do this, go to ‘Update my Information’ (in the upper left-hand corner of the main menu), and click on the Fetch/Validate link next to the ORCID field. This will take you to the ORCID site and allow you to create a new iD or authenticate a pre-existing iD in Editorial Manager. Please see the following video for instructions on linking an ORCID iD to your Editorial Manager account: <https://www.youtube.com/watch?v=_xcclfuvtxQ>

Reply： An ORCID iD has been linked to our Editorial Manager account.

2. Please ensure that you refer to Figure 4 in your text as, if accepted, production will need this reference to link the reader to the figure.

Reply： Figure 4 has been referred in our text in Line 128:A total of 5,795 DEGs were found in "RG vs CL", among which 2,797 DEGs were increased, and 2,998 DEGs were decreased (Fig. 4a). Through the comparative analysis of RG and CL samples, it was found that there were 20,002 DEGs shared both of them. Shared DEGs accounted for about 75% of total DEGs (Fig. 4b).
