# Supplementary material for: Regeneration of duckweed (Lemna turonifera) involves genetic molecular regulation and cyclohexane release
Source: PLoS One. 2022 Jan 6;17(1):e0254265. doi: 10.1371/journal.pone.0254265 (PMC8735602; doi:10.1371/journal.pone.0254265)
Supplement: Attachment — Submitted filename: revise.doc [file pone.0254265.s008.doc]

Dear editor：

Thank you very much for your kindly help in processing the review of our manuscript. We have carefully read these thoughtful comments from you and reviews, and the [minor revision](https://www.editorialmanager.com/pone/javascript:popupLetter(40950885, '{3AD97B27-7B7B-4670-BEA1-29D363EC2690}');) has been done. We appreciate that our research and revision has been affirmed by reviewers. Reviewer 2 said that the authors have satisfactorily addressed all the comments, and I have no more comments. Thus, I endorsed the submission for acceptance in its current form. Reviewer 3 said the manuscript can be acceptable for publication after the minor revision as suggested. Thanks so much for the valuable advice and help from reviewers. We consider all issues mentioned in the reviewers' comments carefully, and made a minor revision as following.

1. For the description of fold change in RNA sequencing data, the 4nd decimal place seems redundant. Please consider to round off to second or third decimal place seems redundant.

Reply: For the description of fold change, the 4nd decimal place has been round off to second decimal place：

Line 107: In this study, AUX/IAA and ARF have been significantly down-regulated, by 13.03 and 3.01 log^2^ fold change, respectively.

Line 115: The expression of ZFP was decreased by 4.04 log2 fold change.

Line 120: Histidine phosphate transfer protein (AHP), which interacts with CRE1 and CKI1, was up-regulated by 2.97 log2 fold change.

Line 124: A-ARR was down-regulated by 4.53 log2 fold change, which might lead to overall up-regulation in cytokinins during callus regeneration.

2. Line 142, please substitute symbol “x” to * in the fulltext.

Reply: we have substituted symbol “x” to *: The peak area of 1, 3-dimethyl benzene in the regenerating callus was 0.84*10^7^

3. Please improve the format of Table 2, 3 and 4.

Reply: The format of Table 2, 3 and 4 have been improved.
